# Efficient Photocatalytic Disinfection of *Escherichia coli* O157:H7 using C_70_-TiO_2_ Hybrid under Visible Light Irradiation

**DOI:** 10.1038/srep25702

**Published:** 2016-05-10

**Authors:** Kai Ouyang, Ke Dai, Sharon L. Walker, Qiaoyun Huang, Xixiang Yin, Peng Cai

**Affiliations:** 1 State Key Laboratory of Agricultural Microbiology, College of Resources and Environment, Huazhong Agricultural University, Wuhan 430070, China; 2 Department of Chemical and Environmental Engineering, University of California, Riverside, California 92521, USA; 3 Jinan Research Academy of Environmental Sciences, Jinan 250014, China

## Abstract

Efficient photocatalytic disinfection of *Escherichia coli* O157:H7 was achieved by using a C_70_ modified TiO_2_ (C_70_-TiO_2_) hybrid as a photocatalyst under visible light (λ > 420 nm) irradiation. Disinfection experiments showed that 73% of *E. coli* O157:H7 died within 2 h with a disinfection rate constant of *k* = 0.01 min^−1^, which is three times that measured for TiO_2_. The mechanism of cell death was investigated by using several scavengers combined with a partition system. The results revealed that diffusing hydroxyl radicals play an important role in the photocatalytically initiated bacterial death, and direct contact between C_70_-TiO_2_ hybrid and bacteria is not indispensable in the photocatalytic disinfection process. Extracellular polymeric substances (EPS) of bacteria have little effect on the disinfection efficiency. Analyses of the inhibitory effect of C_70_-TiO_2_ thin films on *E. coli* O157:H7 showed a decrease of the bacterial concentration from 3 × 10^8^ to 38 cfu mL^−1^ in the solution with C_70_-TiO_2_ thin film in the first 2 h of irradiation and a complete inhibition of the growth of *E. coli* O157:H7 in the later 24 h irradiation.

Millions of people die from diseases transmitted through contaminated water or human excreta every year^1^. As a waterborne pathogenic microorganism, *Escherichia coli* O157:H7 reportedly causes an estimated 73,480 illnesses, 2,168 hospitalizations, and 61 deaths each year in the United States^2^. Reliable, efficient, and environmentally safe disinfection has become an increasing concern throughout the world^3^. Traditional water disinfection methods such as chemical oxidation technologies can require expensive chemicals or costly equipment. Moreover, unwanted oxidation bi-products may be formed in water from chlorination or ozonation processes^4^. Although UV radiation is capable of sustained disinfection, the hazards of intensive and direct use of UV radiation limit its application^5^. Therefore, the development of an efficient and environmental friendly disinfection technology has become an urgent demand.

Since Matsunaga *et al*. discovered the bactericidal activity of TiO_2_ as a photocatalyst for the first time in 1985^6^, many studies related to the bactericidal effect of TiO_2_ photocatalyst have been reported, including the death of bacteria and inactivation of viruses^7^,^8^. However, the potential for the practical application of TiO_2_ is restricted by the broad band gap (3.2 eV) of anatase TiO_2_ and low quantum efficiency^9^. Recently, considerable effort has been taken to address these issues by doping the TiO_2_ with non-metal or metal elements or by coupling with narrow band semiconductors^10^,^11^,^12^. Carbon nanomaterials (fullerene, carbon nanotube, and graphene) are highly valued because of their excellent optical performance, small size effect, and ease in achieving surface functionalization towards coupling with TiO_2_^13^. C_60_ has drawn particular attention of researchers due to its being an excellent electron acceptor with delocalized conjugated structure^14^,^15^,^16^, which can efficiently separate photo-induced charge. C_70_ is a close-shell configuration consisting of 35 bonding molecular orbitals with 70 p-electrons, which can promote efficient electron transfer reduction^17^. Compared with C_60_, C_70_ has higher electron affinity and higher possibility to form anions or free radicals because of the reduced symmetry structure^18^. In addition, C_70_ has a larger photo cross-sectional area, which suggests that a high light harvesting efficiency can be achieved^19^. Latest studies revealed that C_70_-TiO_2_ hybrids show a significant photocatalytic activity for degradation of organic pollutant in visible light region^20^,^21^. The aforementioned reports imply that a high efficient bactericidal activity can be expected from the C_70_-TiO_2_ hybrid.

In the present study, we prepared a C_70_-TiO_2_ hybrid by a hydrothermal reaction process and investigated this material’s capacity for photocatalytic disinfection of *E. coli* O157:H7 under visible light irradiation. The effects of the photocatalyst dosages and the contact modes between C_70_-TiO_2_ hybrid and bacterial cells on the photocatalytic disinfection rates were investigated. Several scavengers for hydroxyl radicals and valence-band holes were applied to examine the roles of the oxidative species in the photocatalytic disinfection of *E. coli* O157:H7. Moreover, the effect of bacterial extracellular polymeric substance (EPS) on the photocatalytic disinfection was also evaluated.

## Results and Discussion

### Crystal Phase Composition, Morphology and Optical Properties of C_70_-TiO_2_ Hybrid

The XRD patterns of TiO_2_ and C_70_-TiO_2_ hybrid are shown in Supplementary Fig. S1. The peaks at 25.4° (101), 37.5° (004), 48.1° (200), 53.8° (105), 54.8° (211), and 62.8° (204) corresponded to the pure anatase phase. It can also be seen that the diffraction peaks of anatase phase in C_70_-TiO_2_ hybrid did not shift with the incorporation of C_70_, indicating that the introduction of C_70_ does not affect the crystalline structure of TiO_2_. Furthermore, no diffraction peak corresponding to C_70_ was observed for the XRD pattern of C_70_-TiO_2_ hybrid, suggesting that C_70_ was well-dispersed in the hybrid^22^.

The representative SEM and HRTEM images of C_70_-TiO_2_ hybrid are displayed in Supplementary Fig. S2. As shown in Supplementary Fig. S2a, TiO_2_ nanoparticles were well dispersed and little aggregated. The surface of hybrid was uneven, which can increase the specific surface area and enhance the photocatalytic activity of C_70_-TiO_2_ hybrid^23^. The presence of C_70_ cannot be identified from the SEM micrograph, which is ascribed to the undetectable size of C_70_ at the present resolution. The lattice fringe of anatase phase can be clearly observed and its spacing is measured to be ca. 0.35 nm (see Supplementary Fig. S2b), which is attributed to the TiO_2_ (101) plane^24^. However, the outer boundary of the sample is distinctly different. The surface of TiO_2_ nanoparticle is surrounded by a noncrystal coverture layer with a thickness of about 1 nm, which is close to the diameter of C_70_ molecule. Thus, the outer layer can be assumed to be C_70_ which is dispersed on the surface of TiO_2_ with a monolayer structure^25^.

The UV-Vis diffuse reflectance spectra of C_70_, TiO_2_ and C_70_-TiO_2_ hybrids are displayed in Supplementary Fig. S3. C_70_ has strong absorption in the ultraviolet region and weak but significant bands in the visible region. The band gap of TiO_2_ is estimated to be 3.17 eV. With the doping of C_70_ into TiO_2_ materials, hybrids exhibited remarkably enhanced light-harvesting performance in the visible light region, indicating that the C_70_-TiO_2_ hybrid can be excited to generate more hole–electron pairs under visible light irradiation due to the C_70_-sensitized activation.

### Comparison of Photocatalytic Disinfection Performance between TiO_2_ and C_70_-TiO_2_ Hybrid

**Figure 1:**
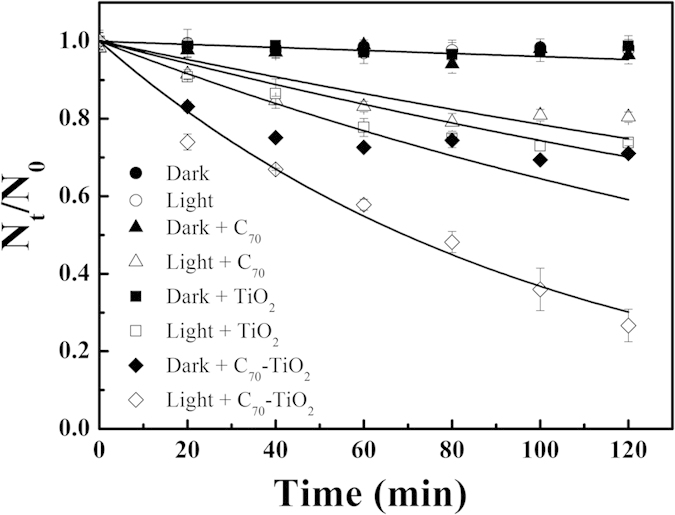
Survival of *E. coli* O157:H7 (0.5 mg mL^−1^) presented as the ratio of live to total cells (N_t_/N_0_) versus the time. Suspensions of cells were exposed to dispersions containing different catalysts (1 mg mL^−1^) in the dark and under visible light irradiation.

*E. coli* O157:H7, a notorious waterborne pathogenic microorganism involved in numerous outbreak events causing foodborne and waterborne diseases, was used to investigate the photocatalytic disinfection performance of C_70_-TiO_2_ hybrid under visible light irradiation. Figure 1 shows the photocatalytic disinfection efficiencies of *E. coli* O157:H7 by C_70_, TiO_2_ and C_70_-TiO_2_ hybrid with the same weight under visible light irradiation (λ ≥ 420 nm). As a comparison, the dark control was carried out with or without the three photocatalysts in the dark. *E. coli* O157:H7 with or without TiO_2_ (or C_70_) in the dark remained mostly viable over a period of 120 min, indicating that TiO_2_ (or C_70_) has no bactericidal effect without light irradiation. However, the survival rate of *E. coli* O157:H7 in the presence of C_70_-TiO_2_ in the dark reduced to 70% after 120 min, indicating that C_70_-TiO_2_ hybrid has physically toxic effects on *E. coli* O157:H7. The light control was also performed with or without the three photocatalysts under visible light irradiation. As shown in Fig. 1, only visible light irradiation without any of the three photocatalysts could not disinfect *E. coli* O157:H7. C_70_ and TiO_2_ have moderate toxic effects on *E. coli* O157:H7 with a disinfection rate of 24% and 30% in 120 min under visible light irradiation, respectively, whereas C_70_-TiO_2_ hybrid kills about 73% of *E. coli* O157:H7 in 120 min, which is significantly higher than that of C_70_ and TiO_2_ under visible light irradiation. The process of disinfection can be fitted with Weibull deactivation function and expressed by equation (1) 26:


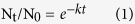


where N_t_/N_0_ represents the number of viable cell divided by the total cell count at time t (min), and *k* is the disinfection rate constant.

Under visible light irradiation, the inactivation rate constant *k* of C_70_-TiO_2_ hybrid was 2.3-fold that of TiO_2_. One possible reason for the higher inactivation efficiency of C_70_-TiO_2_ hybrid is that carbon dots, such as fullerenes (C_60_ and C_70_) and carbon nanotubes, have strong adsorption ability in the visible light region and can increase the visible light adsorption efficiency of C_70_-TiO_2_ hybrid^13^. As mentioned in the result of DRS, C_70_-TiO_2_ composites showed more intensive absorption over the whole visible light region than pure TiO_2_ (Supplementary Fig. S3). Additionally, C_70_ can act as electron traps because of its high electron affinities^17^, which would function as charge separator and enhance the photocatalytic disinfection activity of C_70_-TiO_2_ hybrid.

### Effect of C_70_-TiO_2_ Hybrid Concentration on Photocatalytic Disinfection Performance

**Figure 2:**
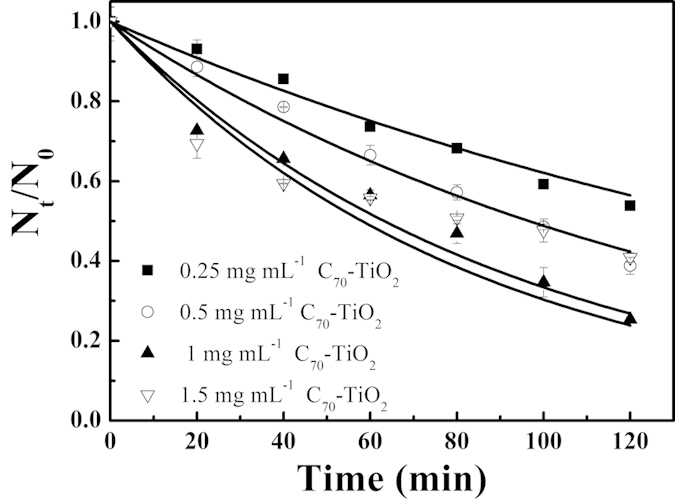
Effect of the catalyst concentration on the disinfection rate of *E. coli* O157:H7.

The disinfection rate of *E. coli* O157:H7 as a function of C_70_-TiO_2_ hybrid concentrations is shown in Fig. 2. With the catalyst dosage increased from 0.25 to 1.00 mg mL^−1^, the deactivation rate of *E. coli* O157:H7 increased, followed by a decrease with the dosage further increased to 1.5 mg mL^−1^. The *k* values of bacteria were 0.005, 0.007, 0.010 and 0.009 min^−1^ corresponding to the catalyst dosages of 0.25, 0.50, 1.00, and 1.50 mg/mL, respectively. These results revealed that the optimum catalyst dosage is 1.0 g L^−1^, suggesting that, with a low dosage, the incident light cannot be completely used by the catalyst, leading to the generation of less active substances. With an increase of the catalyst dosage, more incident light can be absorbed to produce more active substances, which simultaneously can also cause a steady increase of the solution turbidity, resulting in a decline in the utilization of the photons^27^, and finally the reduction of sterilization efficiency.

**Figure 3:**
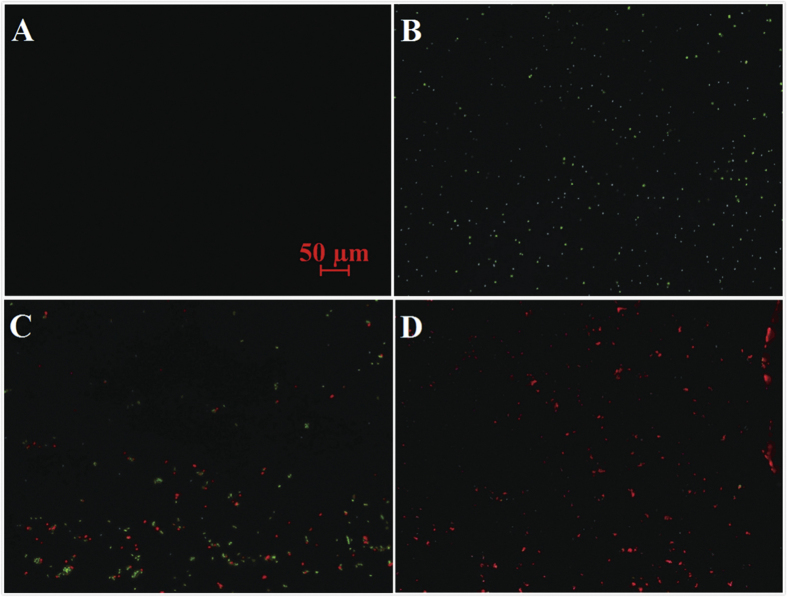
Fluorescence microscopic images of *E. coli* O157:H7 (0.5 mg mL^−1^, 30 mL) treated with C_70_-TiO_2_ hybrid (1 mg mL^−1^) without and under visible light irradiation. (**A**) Only C_70_-TiO_2_ hybrid, (**B**) the mixture of photocatalyst and *E. coli* O157:H7 before irradiation, and after irradiation by visible light for (**C**) 1 h, and (**D**) 2 h.

To further confirm the photocatalytic disinfection performance of C_70_-TiO_2_ hybrid, the fluorescence assays were performed by treating *E. coli* O157:H7 with C_70_-TiO_2_ hybrid without or under visible light irradiation (Fig. 3). The control experiment revealed that C_70_-TiO_2_ hybrid has no color fluorescence (Fig. 3a) and cannot obstruct the observation on bacteria. The bacteria treated only with C_70_-TiO_2_ hybrid can also be observed to exhibit intense green fluorescence (Fig. 3b). In the presence of C_70_-TiO_2_ hybrid, after 1 h irradiation by visible light, nearly half of the cells showed red fluorescence (Fig. 3c), and after 2 h irradiation, almost no living *E. coli* O157:H7 can be observed (Fig. 3d).

Since the Live/Dead BacLight stain only shows the integrity of the cell walls of *E. coli* O157:H7^28^, which cannot demonstrate the culturability of *E. coli* O157:H7. So we used plate count to investigate the survival rate of *E. coli* O157:H7 in the photocatalytic disinfection process. The results demonstrate that the cell culturability of *E. coli* O157:H7 decreased with the extension of irradiation time and was significantly lower than the result measured by the corresponding fluorescent count (see Supplementary Fig. S4). Actually, plate count is not a suitable method to determine the survival rate of the bacteria mixed with other materials. In the process of photocatalytic disinfection, the bacterial cell walls were damaged first, suggesting that the fluorescent staining technique is a direct and accurate method, and thus it was used in the following experiment.

### Photocatalytic Disinfection Mechanism

**Figure 4:**
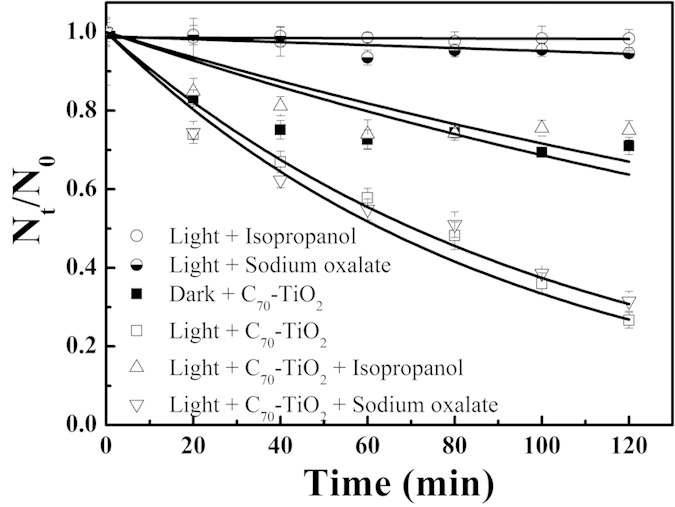
Photocatalytic disinfection of *E. coli* O157:H7 using C_70_-TiO_2_ hybrid with different scavengers (0.3 mol L^−1^ isopropanol, 3.0 × 10^−5^ mol L^−1^ sodium oxalate) under visible light irradiation.

It is well accepted that valence band (VB) holes and conduction band (CB) electrons can be generated when the photocatalyst is irradiated by incident light with a proper wavelength^29^. VB holes can oxidize the H_2_O to produce •OH, and the photogenerated CB electrons can reduce the surface absorbed O_2_ to produce the superoxide radical anion O_2_^−^•. The produced •OH and O_2_^−^• radicals as well as VB holes can disinfect the bacteria. However, there is still a heated argument about which reactive species plays a more significant role in the photocatalytic disinfection process^30^,^31^. Therefore, to address this issue, we used different scavengers individually to remove the specific reactive species. Isopropanol and sodium oxalate were used as the scavengers for •OH and VB holes, respectively. It can be seen from Fig. 4 that the isopropanol and sodium oxalate cannot disinfect the *E. coli* O157:H7 in the absence of photocatalyst. In the presence of sodium oxalate, the photocatalytic disinfection effect of C_70_-TiO_2_ is almost the same as that without the scavenger, indicating that VB holes have almost no action in the disinfection. Interestingly, with the addition of isopropanol to remove •OH in bulk, the photocatalytic disinfection effect of C_70_-TiO_2_ is evidently inhibited (the survival rate of *E. coli* O157:H7 rose from 25% to 74% after 2 h irradiation), indicating that •OH generated by the photocatalyst plays an important role in the disinfection of *E. coli* O157:H7.

**Figure 5:**
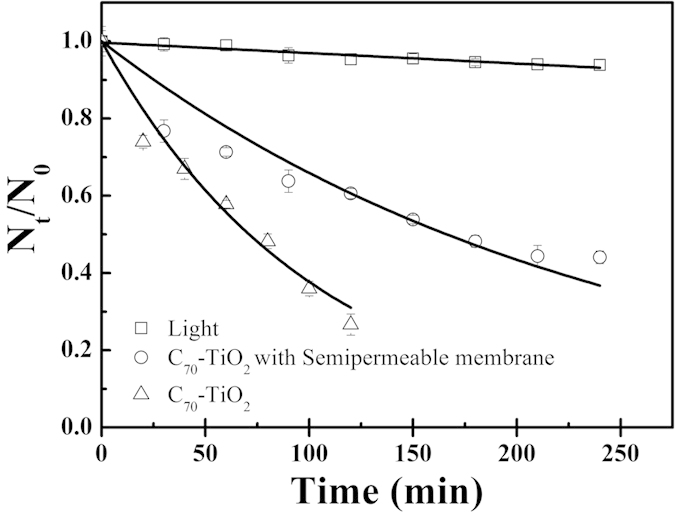
Photocatalytic disinfection of *E. coli* O157:H7 (0.5 mg mL^−1^) with or without a semipermeable membrane in the suspension of 1 mg mL^−1^ C_70_-TiO_2_ hybrid.

It is reported that isopropanol can only capture the diffusing •OH in bulk due to its low affinity to the surface of semiconductors in aqueous media^32^. To further investigate the role of diffusing •OH in the photocatalytic disinfection, we separated *E. coli* O157:H7 and C_70_-TiO_2_ hybrid by using a semipermeable membrane, which only allows water and diffusing •OH to pass through. A suspension of *E. coli* O157:H7 was contained in the container of semipermeable membrane and C_70_-TiO_2_ hybrid was dispersed outside of the container. As shown in Fig. 5, the survival rate of *E. coli* O157:H7 in the semipermeable membrane increased from 25% to 60% after 2 h visible-light irradiation, indicating that the photocatalytic disinfection efficiency of *E. coli* O157:H7 in the separated system (Fig. 5) is much lower than that in the nonseparated system. This phenomenon indicates that the diffusing •OH is quite important to the disinfection of *E. coli* O157:H7, which is consistent with the result of Kubacka’s study. Kubacka *et al*. recently reported a detailed genetic investigation into the mechanism of TiO_2_ photocatalysis and concluded that extensive radical induced cell wall modifications are the main factor responsible for the high biocidal performance of TiO_2_-based nanomaterials^33^. In addition, the above result of our study also suggests that the direct contact between C_70_-TiO_2_ hybrid and *E. coli* O157:H7 is not indispensable for the photocatalytic disinfection.

**Figure 6:**
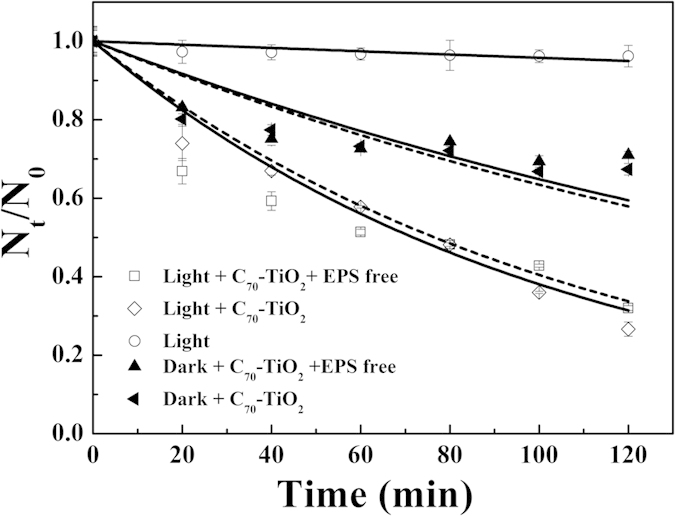
Photocatalytic disinfection of *E. coli* O157:H7 using C_70_-TiO_2_ hybrid as a function of cells with full EPS or partially extracted EPS.

As demonstrated in Zhang’s research^34^, the destruction of the outer membrane of *E. coli* K-12 was the initial step in the photocatalytic disinfection. To verify the role of bacterial extracellular polymeric substance (EPS) — an important reactive component associated with bacterial cell walls in the photocatalytic disinfection, we removed EPS from *E. coli* O157:H7 by using cationic exchange resin (CER)^35^ and tested the survival rate of EPS-free *E. coli* O157:H7 in the photocatalytic disinfection. As shown in Fig. 6, there is almost no difference between the disinfection rates of *E. coli* O157:H7 and EPS-free *E. coli* O157:H7 either under visible light irradiation or in the dark. As mentioned above, the attack by •OH contributes a large part to the disinfection of *E. coli* O157:H7. As an electrophilic species, •OH usually does not attack EPS which mainly contains a high molecular weight polymer such as polysaccharides, proteins, and nucleic acids. Only if the amount of •OH is increased to a certain level can the unselective attack on both EPS and cell wall of *E. coli* O157:H7 by •OH be observed.

**Figure 7:**
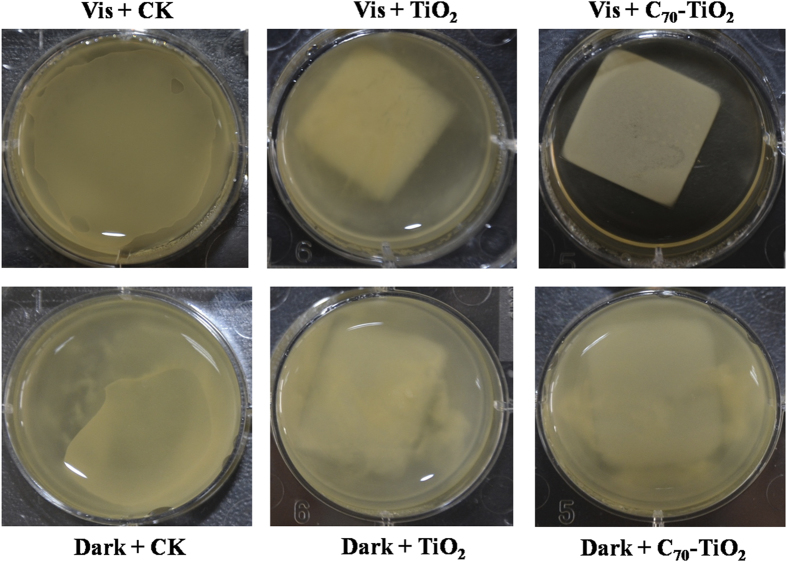
Inhibitory effects of TiO_2_ and C_70_-TiO_2_ thin films on the formation of *E. coli* O157:H7 biofilm in the dark and under visible light irradiation. Where the square piece is TiO_2_ or C_70_-TiO_2_ thin film, CK refers to control experiment without TiO_2_ and C_70_-TiO_2_ thin film.

In real applications of photocatalytic disinfection, the photocatalysts are usually immobilized to facilitate the recovery. Therefore, the photocatalytic disinfection efficiency of C_70_-TiO_2_ thin film was examined. The C_70_-TiO_2_ thin films were prepared by dispersion, coating and natural drying. The inhibitory effect of C_70_-TiO_2_ thin films on *E. coli* O157:H7 was investigated. Figure 7 shows the growth status of *E. coli* O157:H7 under different conditions. In the absence of photocatalyst, LB medium in the orifice plate becomes very turbid whether or not in the dark, resulting from the massive growth of bacteria. Furthermore, *E. coli* O157:H7 retains its viability in the solution containing TiO_2_ thin film or C_70_-TiO_2_ thin film without visible light irradiation. However, the LB medium solution containing visible-light-irradiated C_70_-TiO_2_ thin film in the orifice plate is as limpid as before the treatment. The concentration of bacteria dropped from 3 × 10^8^ to 38 cfu/mL in the solution with C_70_-TiO_2_ thin film after 2 h irradiation and the growth of *E. coli* O157:H7 was completely inhibited in the later 24 h irradiation. These experimental results manifested the efficient and durable bactericidal activity of C_70_-TiO_2_ thin film under visible light irradiation.

To evaluate the reusability of C_70_-TiO_2_ thin film, the used thin film was collected from the LB medium after the disinfection experiment, washed with distilled water, and dried for the next cycle. It is noteworthy that after four cycle tests for a total of 100 hours, the C_70_-TiO_2_ thin film showed no obvious loss of photocatalytic disinfection activity after the 100 h cycled test (see Supplementary Fig. S5), indicating that C_70_-TiO_2_ hybrid is a highly stable photocatalyst for the disinfection of *E. coli* O157:H7 under visible light irradiation. The overall results suggest that C_70_-TiO_2_ hybrid is stable and reusable as a promising efficient photocatalyst for inactivation of waterborne pathogenic microorganisms.

## Materials and Methods

### Preparation of C_70_-TiO_2_ Hybrid

C_70_ (purity < 99.0 wt%) was purchased from Puyang Yongxin Fullerene Technology Co., Ltd. (CAS, China) and modified as follows: C_70_ was suspended in 35 mL of deionized water and concentrated nitric acid (volume ratio of water: HNO_3_ = 7:1), followed by heat stirring for 2 h, and washing with deionized water until the pH value of the supernatant became neutral.

C_70_-TiO_2_ hybrid was prepared using the hydrothermal method. Titanium sulfate, cetyltrimethylammonium bromide (CTAB) and deionized water (mass ratio of Ti(SO_4_)_2_:CTAB:water = 1:0.12:100) were stirred evenly with the activated C_70_. The weight ratio of C_70_ and Ti(SO_4_)_2_ was controlled at 1:27.3, which is equal to a weight ratio of 18:82 for the C_70_ and TiO_2_ (18 wt% C_70_-TiO_2_). Next, the reaction mixture was transferred into a 50 mL Teflon lined stainless steel autoclave and underwent hydrothermal treatment at 100 °C for 72 h. After cooling to ambient temperature, the mixture was centrifuged and supplemented with deionized water and ethanol (1:1 v/v), followed by an ion exchange treatment by mixing the as-prepared sample with an excess sodium chloride under stirring for 24 h. Subsequently, the sample was washed 2 to 3 times separately with deionized water and ethanol, and dried at 90 °C for 10 h. Finally, the mixture was calcined from room temperature to 400 °C, held for 2 h, and then ground to uniformity^21^.

### Characterization of the C_70_-TiO_2_ Hybrid

X-ray diffraction (XRD) patterns were analyzed from an X-ray diffractometer (D8 advance, Bruker Inc., Germany) with Cu Kα radiation source at 35 kV. The surface state and structure of C_70_-TiO_2_ hybrid were observed by scanning electron microscopy (SEM) (JSM-6700F JOEL, Japan) and transmission electron microscopy (TEM) (JEM-2100F, JEOL, Japan). UV-Vis diffused reflectance spectroscopy of the power solids was carried out using a UV-Vis spectrophotometer (UV-3100, Shimadzu Inc., Japan).

### Culture and Collection of *E. coli* O157:H7

Prior to each experiment, a pre-culture was prepared by incubating a sample of *E. coli* O157:H7 at 37 °C overnight in Luria-Bertani (LB) broth. The overnight pre-culture was added to 250 mL of LB broth in a 500 mL Erlenmeyer flask. The culture was incubated at 37 °C for 3.5 h at 150 rpm until mid-exponential growth phase was reached. Cells were harvested by centrifugation at 4000 rpm for 10 min. The growth medium was decanted and the pellet was resuspended in 10 mM KCl and the centrifugation repeated. Centrifugation and resuspension were repeated one additional time to remove traces of growth media and metabolites. The final pellet was resuspended in 10 mM KCl, and the suspensions were diluted to the desired final concentration of 10 mg mL^−1^.

### Preparation of EPS-free *E. coli* O157:H7

EPS-free *E. coli* O157:H7 was obtained through the treatment by cationic exchange resin (CER)^36^. Specifically, 30 mL of suspension containing about 0.6 g of bacterial cells (wet weight) was mixed with 30 g of CER (732), which was rinsed with ultrapure water for several times prior to use. After thorough mixing on a magnetic stirrer at 4 °C for 24 h, the suspension was allowed to settle and the CER was gathered on the bottom. The treated cell suspension in the aliquots phase was washed three times in ultrapure water in order to separate any remaining CER.

### Photocatalytic Disinfection of *E. coli* O157:H7

A suspension of C_70_-TiO_2_ particles and *E. coli* O157:H7 cells was added into a double walled borosilicate immersion well of 43 mm outer diameter with inlet and outlet for water circulation to keep the temperature of mixed suspension at 25 °C, and the reaction mixture was stirred with a magnetic stirrer. The photocatalyst concentration and cell density in the suspension were adjusted to 10 mg L^−1^ and 0.5 mg mL^−1^, respectively. The radiation source was a 300 W Xenon lamp (PLS SXE300C, Beijing Perfect Light inc., China) with a filter (λ > 420 nm). The light intensity was fixed at 600 μW cm^−2^. After the photocatalytic disinfection treatment, an aliquot of the reaction solution was sampled. The samples were fluorescently stained with the LIVE/DEAD BacLight bacterial viability kit (L13152, Molecular Probes, Inc., Eugene, OR). After incubation in the dark for 15 min, the samples were examined using a fluorescence microscope (Zeiss Imager A1, Germany). The living cells showed green fluorescence under emitted light 460–500 nm and the dead cells showed red fluorescence under emitted light 510–560 nm. The survival ratio of the *E. coli* O157:H7 was estimated by the number of viable cells divided by the total cell count. As a comparison, a dark control (without light irradiation and photocatalyst), a light control (light irradiation alone without the photocatalyst), and a negative control (without the photocatalyst or light irradiation) were also conducted. All the photocatalytic bacterial disinfection experiments were performed in triplicate.

To study whether direct contact between photocatalyst and *E. coli* O157:H7 is important for disinfection, a partition system^37^ was used to separate *E. coli* O157:H7 from the surface of photocatalyst. *E. coli* O157:H7 suspension was pipetted into a semipermeable container (semipermeable membrane), and the photocatalyst suspension was maintained outside of the membrane and stirred continuously to keep the photocatalyst evenly distributed in the solution. At different intervals, aliquots of the cells inside the membrane were sampled, and the number of viable cells in the samples was determined as described above.

The scavenger experiments were carried out by adding individual scavenger to the photocatalytic reaction system. Isopropanol and sodium oxalate were used as the scavengers for •OH and VB holes, respectively^10^.

## Additional Information

**How to cite this article**: Ouyang, K. *et al*. Efficient Photocatalytic Disinfection of *Escherichia coli* O157:H7 using C_70_-TiO_2_ Hybrid under Visible Light Irradiation. *Sci. Rep*. **6**, 25702; doi: 10.1038/srep25702 (2016).

## Supplementary Material

Supplementary Information
