# Supplementary material for: Efficient Photocatalytic Disinfection of Escherichia coli O157:H7 using C70-TiO2 Hybrid under Visible Light Irradiation
Source: Sci Rep. 2016 May 10;6:25702. doi: 10.1038/srep25702 (PMC4861983; doi:10.1038/srep25702)
Supplement: Supplementary Information [file srep25702-s1.doc]

**Supplementary Information for**

**Efficient Photocatalytic Disinfection of *Escherichia coli* O157:H7 using C_70_-TiO_2_ Hybrid under Visible Light Irradiation**

Kai Ouyang^a1^, Ke Dai^a1^, Sharon L. Walker^b^, Qiaoyun Huang^a^, XixiangYin^c^, Peng Caia[[1]](#footnote-2)*

^a^ *State Key Laboratory of Agricultural Microbiology, College of Resources and Environment, Huazhong Agricultural University, Wuhan 430070, China*

^b^ *Department of Chemical and Environmental Engineering, University of California, Riverside,* *California 92521, USA*

^c^ *Jinan Research Academy of Environmental Sciences，Jinan 250014，China*

^*^ Corresponding author. Tel.: +86-27-87671033. Fax: +86-27-87280670.

E-mail address: cp@mail.hzau.edu.cn (P. Cai).

^1^ These authors contributed equally to this work.

**Supplementary Figure Legends**

Figure S1. XRD patterns of TiO_2_ and C_70_-TiO_2_ hybrid.

Figure S2. SEM (a) and High resolution TEM (b) images of the C_70_-TiO_2_ hybrid.

Figure S3. UV-Vis DRS patterns of C_70_, TiO_2_ and C_70_–TiO_2_.

Figure S4. Comparison of the survival ratios calculated by plate count and fluorescence microscopy for *E. coli* O157:H7 using C_70_-TiO_2_ hybrid as the photocatalyst.

Figure S5. Repeated experiments on the photocatalytic disinfection of *E. coli* O157:H7 with C_70_-TiO_2_ film.

Figure S1 XRD patterns of TiO_2_ and C_70_-TiO_2_ hybrid.


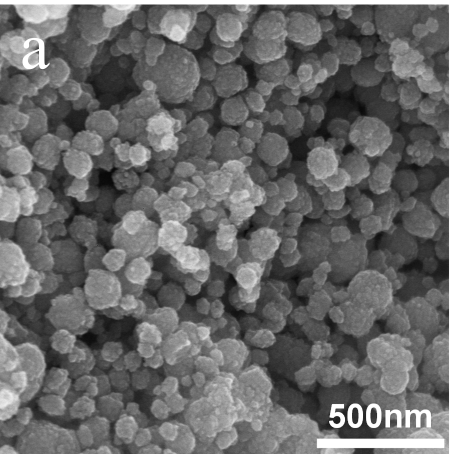

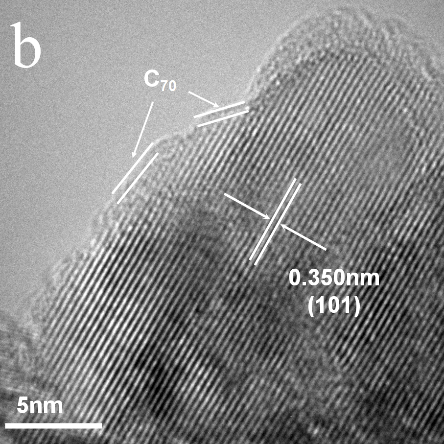


Figure S2 SEM (a) and High resolution TEM (b) images of the C_70_-TiO_2_ hybrid.

Figure S3 UV-Vis DRS patterns of C_70_, TiO_2_ and C_70_–TiO_2_.

Figure S4 Comparison of the survival ratios calculated by plate count and fluorescence microscopy for *E. coli* O157:H7 using C_70_-TiO_2_ hybrid as the photocatalyst.

Figure S5 Repeated experiments on the photocatalytic disinfection of *E. coli* O157:H7 with C_70_-TiO_2_ film.

1. ^^ [↑](#footnote-ref-2)
